# Molecular epidemiological association between antimicrobial resistance characteristics and type III secretion system in carbapenem-resistant *Pseudomonas aeruginosa* from lower respiratory tract infections

**DOI:** 10.3389/fcimb.2026.1757868

**Published:** 2026-02-17

**Authors:** Yun Zhou, Yanzi Chang, Chen Huang, Ximing Wang, Feng Wang, Hong Li

**Affiliations:** 1 Department of Clinical Laboratory, The Affiliated Lihuili Hospital of Ningbo University, Ningbo, Zhejiang, China; 2 Department of Scientific Research, The Affiliated Lihuili Hospital of Ningbo University, Ningbo, Zhejiang, China; 3 Department of Pulmonary and Critical Care Medicine, The Affiliated Lihuili Hospital of Ningbo University, Ningbo, Zhejiang, China; 4 Basic Medical Sciences and Forensic Medicine, Hangzhou Medical College, Hangzhou, Zhejiang, China; 5 Department of Hepatobiliary and Pancreatic Surgery, The Affiliated Lihuili Hospital of Ningbo University, Ningbo, Zhejiang, China

**Keywords:** Bla_KPC-3_, CRPA, *ExoS*, *ExoU*, ST1076-O11

## Abstract

**Objective:**

Objective: This study investigated the antimicrobial resistance profile of carbapenem-resistant *Pseudomonas aeruginosa* (CRPA) and its association with the T3SS, aiming to provide evidence for managing highly resistant and virulent CRPA and to guide the optimization of infection control strategies.

**Methods:**

Retrospective cohort study was performed with CRPA lower respiratory tract infection from a tertiary hospital (July, 2021, and May, 2025). All strains underwent whole-genome sequencing (WGS), antimicrobial susceptibility testing(AST); selected isolates’ virulence were further evaluated using galleria mellonella infection assays.

**Results:**

Among 106 patients caused by CRPA infections, the majority were male (73.6%), with a median age of 69.5 years and median hospital stay of 26.5 days. Prior antibiotic exposure included piperacillin/tazobactam (57.5%) and carbapenems (49.1%). AST revealed high resistance to piperacillin/tazobactam, but favorable susceptibility to ceftazidime/avibactam and tobramycin, with no polymyxin B resistance. Multidrug resistance occurred in 49.1% of isolates, and 28.3% were difficult-to-treat *Pseudomonas aeruginosa* (DTR-PA). The *exoU* and *exoS* genes were detected in 28.3% and 64.2% of strains, respectively, with 7.5% co-detection. *ExoU*+/*exoS*- strains showed significantly higher resistance to ceftazidime, cefepime, and piperacillin/tazobactam. All isolates had *oprD* mutations. Carbapenemase genes were found in 21.7%, primarily *bla*_KPC-3._*ExoU+/exoS-* strains were linked to *bla_KPC-3_* and *bla*_OXA-50_ subtypes(OXA-488,OXA-1032), while *exoU-/exoS+* strains associated with *bla_OXA-486_* and *bla_OXA-904_*. Among 59 sequence types, ST1076 (13.2%) and ST491 (10.4%) dominated, with serotypes O11 (38.7%) and O6 (25.5%) prevalent. Six international high-risk clones included ST233, ST244, and ST357. Phylogenetically, phylogroup A (*exoU*-/*exoS*+, e.g., PAO1) was larger, and phylogroup B (*exoU*+/*exoS*-, e.g., PA14) included highly virulent strains. *ExoU*+/*exoS*- strains grouped in ST1076 and ST357, and *exoU*+/*exoS*+ in ST463. The survival of *galleria mellonella* further indicated that carrying the *exoU* may cause a higher lethality rate.

**Conclusions:**

*ExoU*+/*exoS*- strains were strongly associated with resistance genes like *bla*_KPC-3_,*bla*_OXA-488_, *bla*_OXA-1032_, ICU admission and exhibited a more pronounced drug-resistant phenotype. Potential high-risk clones such as ST1076-O11 and ST463-O4 display both extensive resistance and high virulence, underscoring the clinical and public health importance of enhanced surveillance and effective containment.

## 1 Introduction

*Pseudomonas aeruginosa* is one of the most common opportunistic pathogens. Despite a slight decrease in its isolation rate in recent years, it continues to be a significant etiological agent of healthcare-associated lower respiratory tract infections (Weiner et al., 2016). Carbapenem-resistant *Pseudomonas aeruginosa* (CRPA) presents considerable therapeutic challenges in clinical settings due to its inherent resistance mechanisms and strong environmental adaptability. According to data from the European Antimicrobial Resistance Surveillance Network (EARS-Net), the detection rate of CRPA was 18.1% in 2021. In the United States, the isolation rate of CRPA varied between 10% and 30% (Tenover et al., 2022), while in China it was 15.2% (Karlowsky et al., 2021). The 30-day overall mortality rate attributed to CRPA infection is above 18.1%, and there were significant geographical and infection-type differences in mortality; specifically, the mortality rate for lower respiratory tract infections attributed to CRPA can be as high as 19% (Reyes et al., 2023). Considering its significant threat to global health, the World Health Organization (WHO) reaffirmed the classification of CRPA as a critical priority pathogen in 2024 (Sati et al., 2025). Moreover, for better classification of the resistance profile of *Pseudomonas aeruginosa*, the additional new class of difficult-to-treat resistant Pseudomonas aeruginosa (DTR-PA) was introduced in 2018 (Kadri et al., 2018). DTR-PA is defined by strains that exhibit non-susceptibility to all of the following antimicrobial agents: piperacillin-tazobactam, ceftazidime, cefepime, aztreonam, meropenem, imipenem-cilastatin, ciprofloxacin, and levofloxacin (Tamma et al., 2024). The detection rate of DTR-PA has increased in recent years, presenting a significant threat to human health (Wu et al., 2024).

The resistance mechanisms of CRPA are multifaceted and can be primarily classified into three categories: β-lactamase production or overexpression, mutations in outer membrane proteins, and overexpression of efflux pump systems. Among these, non-enzymatic carbapenem resistance mechanisms are relatively prevalent (Falcone et al., 2024). Additionally, virulence genes are crucial in the pathogenicity of CRPA, which possesses various virulence factors such as lipopolysaccharide (LPS), quorum sensing, two-component systems, type VI secretion systems (T6SS), outer membrane vesicles, and the CRISPR-Cas system along with its regulatory mechanisms. These virulence factors increase bacterial pathogenicity via multiple mechanisms and cause harm to host cells (Letizia et al., 2025). Within the type III secretion system (T3SS), four key effectors—*exoU*, *exoT*, *exoY*, and *exoS*—play significant roles in bacterial pathogenesis by enabling the translocation of toxic proteins into host cells (Hu et al., 2013; Hardy et al., 2022; Nolasco-Romero et al., 2024). *ExoS* and *exoU* are considered as the major contributors in the pathogenicity of *Pseudomonas aeruginosa*. Notably, *exoS*^+^/*exoU*^+^ strains have been reported in 19 countries or regions worldwide (Song et al., 2023). A multicenter study conducted in China revealed that the majority of *exoU*+ strains display high virulence, and clonal clusters of ST463 *exoU*+ *Pseudomonas aeruginosa* were identified in southeastern China (Zhu et al., 2023). Such results emphasize the importance of considering local epidemiology since there is geographical diversity not only in resistance mechanisms but also in high-virulence genotypes.

Despite the growing recognition of the interplay between bacterial virulence and antimicrobial resistance, the epidemiological association between the resistance profile of CRPA and T3SS virulence genotypes in lower respiratory tract infections is not well elucidated. In order to further investigate the correlation between them, this study conducted WGS analysis on 106 clinical CRPA isolates obtained from a tertiary hospital in Ningbo, collected between July 2021 and May 2025. It also studied the association among T3SS virulence gene presence, bacterial virulence, antibiotic resistance, and molecular epidemiological patterns. This study aim to provide evidence for the optimization of dual surveillance strategies focused on high-virulence, DTR-PA strains.

## 2 Materials and methods

### 2.1 Samples and bacterial strains

From July 1,2021 to May 31,2025,we enrolled 106 patients with a first CRPA-positive lower respirary tract infection and new clinical data from Ningbo medical center Lihuili hospital in China. A total of 106 CRPA strains were identified by VITEK MS. *Escherichia coli* ATCC 25922, *Escherichia coli* ATCC 8739, *Pseudomonas aeruginosa* ATCC 27853 and PAO1 were used as quality control strains.

### 2.2 Equipment and reagents

Columbia blood agar plates were purchased from Zhengzhou Antu Biotechnology Co., Ltd. (Zhengzhou, China). Customized antimicrobial susceptibility testing (AST) plates against *Pseudomonas aeruginosa* were acquired from Wenzhou Kangtai Biotechnology Co., Ltd. (Wenzhou, China). A biological safety cabinet was bought from Haier Biomedical Co., Ltd. (Qingdao, China). A constant-temperature incubator was obtained from Shanghai Yiheng Scientific Instruments Co., Ltd. (Shanghai, China). A mass spectrometer for microbial identification was purchased from bioMérieux (Marcy-l’Étoile, France). A Covaris ultrasonic cell disruptor was obtained from Covaris, Inc. (Woburn, MA, USA). The Agilent Bioanalyzer 5400 system was purchased from Agilent Technologies, Inc. (Santa Clara, CA, USA). The Illumina NovaSeq X Plus Series (paired-end 150 bp, PE150) was obtained from Illumina, Inc. (San Diego, CA, USA).

### 2.3 Strain resuscitation, identification, and AST

After 106 CRPA strains were collected from a -80°C freezer, three-zone streaking was conducted on Columbia blood agar plates to isolate individual colonies, which were subsequently incubated overnight at 35°C in a constant-temperature incubator. The colonies were subcultured to purify them following the growth of individual colonies. Colony samples from the blood agar plates were scraped and identified using a VITEK mass spectrometer. All strains were tested for minimum inhibitory concentrations (MICs) of various antibacterial agents following the guidelines of the *Pseudomonas aeruginosa* customized AST plate using the microbroth dilution method. The agents tested included ceftazidime, cefepime, piperacillin/tazobactam, cefoperazone/sulbactam, tobramycin, ciprofloxacin, levofloxacin, aztreonam, imipenem, meropenem, ceftazidime/avibactam, and polymyxin B. AST was analyzed in accordance with the CLSI M100-Ed35 Performance Standards for Antimicrobial Susceptibility Testing (AST). The breakpoints for cefoperazone were utilized for cefoperazone/sulbactam, while those for polymyxin B adhered to EUCAST criteria (European Committee on Antimicrobial Susceptibility Testing, 2025).

### 2.4 Next-generation WGS

DNA was extracted and purified from the strains utilizing the STE method for WGS. DNA libraries were constructed using the Illumina NEBNext Ultra II FS DNA Library Prep Kit (New England Biolabs, Ipswich, MA, USA) following the manufacturer’s instructions. After library construction, the insert size of the libraries was assessed using the Agilent 5400 system (AATI). When the insert size met the expected criteria, the effective concentration (1.5 nM) of the libraries was accurately quantified using the qPCR method to verify library quality. Subsequent paired-end sequencing was conducted using 150 base pairs on the Illumina NovaSeq platform. This method followed the fundamental principles of classic protocols (Maniatis and Sambrook, 1982; Sambrook, 2001) and incorporated several optimized protocols (Bairoch and Apweiler, 2000; Nguyen et al., 2011; Rehm et al., 2013). All the above procedures were conducted by Beijing Novogene Bioinformatics Technology Co., Ltd. The genomes of 106 CRPA strains have been deposited in the Genome Sequence Archive under National Genomics Data Center accession CRA033966) accessible at https://ngdc.cncb.ac.cn/gsa (CNCB-NGDC Members and Partners, 2025; Zhang et al., 2025).

### 2.5 Bioinformatics analysis workflow

CRPA genome assembly and annotation, including serotypes and MLST types, were conducted using the online visualization platform tailored for microbial genome analysis available at https://cloud.mimazi.net. The Fastp v0.23.1 software (key parameters: minimum read length 80nt, minimum quality value 20) was utilized for sequencing data quality control and statistics. Jellyfish v2.3.1 (key parameter: kmer 17) was used to complete the assessment of genomic K-mer frequency. SAMtools, BWA, and BED tools with default parameter settings were employed for GC-Depth distribution analysis. SPAdes v4.2.0 (key parameters: kmer 35-115) was utilized for sequence assembly, optimizing sequences with multiple Kmer parameters to achieve the most favorable assembly results. Drug resistance genes were carried out simultaneously referenced from Resfinder v4.7.2 (key parameters: coverage ≥ 0.6, identity ≥ 0.8) and the CARD database (key parameters: identity ≥ 80%; coverage ≥ 50%; evalue ≤ 1e-5). Additionally, the results were cross-validated and refined using the Pathogen database (https://www.ncbi.nlm.nih.gov/pathogens/). The ST number was assigned by searches against the tool of MLST analysis (batch annotation) v1.0. Serotyping was accomplished by the tool of serotyping *P. aeruginosa* (PAst v3) v1.0. We aligned these sequences against the genome sequences of *P. aeruginosa* PAO1 using the “snippy” tool (snippy v4.6.0, tar v1.32, and Galaxy v4.6.0+galaxy0) within the online analysis system at https://usegalaxy.cn/ (Seemann, 2015). This alignment helped to identify any variations or mutations in these specific genes (*oprD*, *mexR*, *mexB*, *nalC*, and *nalD*) compared to the reference genome.

### 2.6 Phylogenetic tree construction and heatmap analysis of drug-resistant genes

In this study, OrthoFinder version 2.4.0 (Emms D.M. & Kelly S (2019)., Genome Biology 20:238) was employed for homologous gene analysis with default parameters. Through the analysis of homologous genes, single-copy orthologous genes were identified across all samples. Each single-copy orthologous gene underwent multiple sequence alignment through MUSCLE v3.8.31 (http://www.drive5.com/muscle), and the aligned sequences were trimmed with GBLOCKS 0.91b to remove low-quality alignment results at both ends. All aligned single-copy orthologous sequences were subsequently concatenated in the same order. Finally, FastTree (http://www.microbesonline.org/fasttree/) was used to construct a phylogenetic tree based on the Maximum Likelihood (ML) method. The enhancement of sample information in the phylogenetic tree was conducted using the iTOL web server (https://itol.embl.de), while the heatmap of drug-resistant genes was produced with TBtools-II v2.360. The combination of these two elements was achieved through Adobe Illustrator.

### 2.7 Toxicity assay of *Galleria mellonella*

To evaluate the virulence levels of these strains (Tsai et al., 2016), healthy cream-colored galleria mellonella larvae with similar body weights (150–200 mg) were selected, and the ages of the larvae were standardized. After resuscitating the strains, respective *Pseudomonas aeruginosa* PAO1 suspensions with concentrations of 10×10⁶ CFU/mL, 10×10⁵ CFU/mL, and 10×10⁴ CFU/mL were prepared. According to the result of the preliminary experiment, a dose of 1×10⁴ CFU/mL was chosen to infect *Galleria mellonella* larvae. The 10 μL of prepared bacterial suspensions from various clinical strains were injected into the larvae via the last left abdominal proleg, administered using a disposable insulin syringe (U100-31G8mm). The injected larvae were then placed in a constant temperature incubator at 37°C, and their survival status was monitored every 2 h. The experiment lasted for 24 h, and 10 larvae were used in each group. Larvae were considered dead when they failed to respond to repeated external stimuli. In this experiment, *Pseudomonas aeruginosa* PAO1 was used as a median-virulence control, and untreated larvae and larvae injected with sterile PBS served as blank controls. Survival curves were generated by averaging the results of 3 replicates. The *P* value of Kaplan Meier’analysis was calculated with log-rank test.

### 2.8 Statistical analysis

Statistical analysis was performed using SPSS 25.0 software. The chi-square test and Fisher’s exact test were applied for comparation, and a *P* < 0.05 was considered statistically significant. To control the false positive risk brought by multiple comparisons, the false discovery rate (FDR) correction was applied to all the P values obtained from the tests.

## 3 Results

### 3.1 Clinical data of 106 patients

This study enrolled 106 patients with lower respiratory tract infections due to *Pseudomonas aeruginosa* from July 2021 to May 2025. Among them, there were 78 males and 28 females, with a median age of 69.5 years and an interquartile range (IQR = 61.0–81.0); the median length of hospital stay was 26.5 days, with an interquartile range (IQR = 17.8–44.0); patients were mainly from ICU (n = 53, 50.0%), EICU (n = 21, 19.8%), and the Respiratory Critical Care Department (n = 16, 15.1%). Before the identification of *Pseudomonas aeruginosa*, all but 3 patients had received antibiotic therapy: 34 patients had received at least three or more classes of antibacterial agents, 61 patients (57.5%) had received piperacillin/tazobactam, and 52 patients (49.1%) had been administered carbapenem antibiotics (**Table 1**).

**Table 1:** Clinical data of 106 patients during this study.

Item	Number of patients	Percetage(%)
Sex		
Male	78	73.6
Female	28	26.4
Age(Years)		
18-64	36	34.0
>=65	70	66.0
Department		
ICU	53	50.0
EICU	21	19.8
Department of Pulmonary Critical Care Medicine	16	15.1
Department of Neurosurgery	6	5.7
Department of Geriatrics	5	4.7
Others	5	4.7
Antimicrobial exposure		
Cabapenems	52	49.1
Cefoperazone/sulbactam	18	17.0
Piperacillin/tazobatam	61	57.5
Ceftazidime-avibactam	8	7.5
Cephalosporins	18	17.0
Fluoroquinolones	10	9.4
Aminoglycosides	1	0.9
Macrolides	4	3.8
Polymyxin B	4	3.8
Tigecycline/Omadacycline/Eravacycline	10	9.4
Vancomycin/linezolid	19	17.9
Antifungal agents	5	4.7
Unused	3	2.8

### 3.2 AST results and their correlation with virulence types of T3SS

AST results revealed that 106 strains of CRPA exhibited significant variations in resistance to clinically commonly used antimicrobial agents. With the exception of carbapenems (the MIC_50_ and MIC_90_ of imipenem were 8 μg/mL and >128 μg/mL, respectively; those of meropenem were 8 μg/mL and >128 μg/mL, respectively), these strains exhibited the highest resistance rates to piperacillin/tazobactam, followed by levofloxacin and ceftazidime. In contrast, they displayed good susceptibility to ceftazidime/avibactam and tobramycin, and no polymyxin B-resistant strains were detected. Notably, according to these criteria (Magiorakos et al., 2012; Tamma et al., 2024), 52 strains (49.1%) were identified as multidrug-resistant *Pseudomonas aeruginosa* (MDR-PA), and 30 strains (28.3%) as extensively DTR-PA(**Supplementary Tables 1**, **2**).

The detection rates of the *exoU* and *exoS* genes were 28.3% (30/106) and 64.2% (68/106), respectively, with a co-detection rate of 7.5% (8/106) for both genes. *ExoU*+/*exoS*- strains demonstrated a significant increase in resistance to ceftazidime (*P* = 0.001), cefepime (*P* = 0.004), and piperacillin/tazobactam (*P* < 0.001) when compared to *exoU*-/*exoS*+ strain. However, there was no statistically significant association between the virulence genotypes and the resistance phenotypes of the other tested antimicrobial agents (**Table 2**).

**Table 2:** Association between T3SS virulotypes and antimicrobial resistance profiles in carbapenem resistance *Pseudomonas aeruginosa* isolates from lower respiratory tract infections.

Antimicrobial agents	No.(%)of isolates by T3SS virulotypes			
	*exoU*+/*exoS*- (n=30)	*exoU*-/*exoS*+ (n=68)	*exoU*+/*exoS*+ (n=8)	*P* value for comparison between *exoU*+/*exoS*- and *exoU*-/*exoS*+
Ceftazidime	19(63.3)	18(26.5)	3(37.5)	0.001
Cefepime	13(43.3)	11(16.2)	3(37.5)	0.004
Aztreonam	15(50.0)	21(30.1)	3(37.5)	0.070
Tobramycin	0(0.0)	9(13.2)	1(12.5)	0.053
Ciprofloxacin	9(30.0)	17(25.0)	5(62.5)	0.605
Levofloxacin	17(56.7)	25(36.8)	5((62.5)	0.067
Imipenem	30(100.0)	60(88.2)	8(100.0)	0.102
Meropenem	25(83.3)	44(64.7)	5(62.5)	0.063
Piperacillin/tazobactam	22(73.3)	23(33.8)	3(37.5)	<0.001
Cefoperazone/sulbactam	13(43.3)	17(25.0)	3(37.5)	0.070
Ceftazidime/avibactam	3(10.0)	5(7.4)	2(25.0)	0.659
Polymyxin B	0(0.0)	0(0.0)	0(0.0)	/

### 3.3 Molecular mechanisms of acquired resistance in *CRPA*

**Figure 1:**
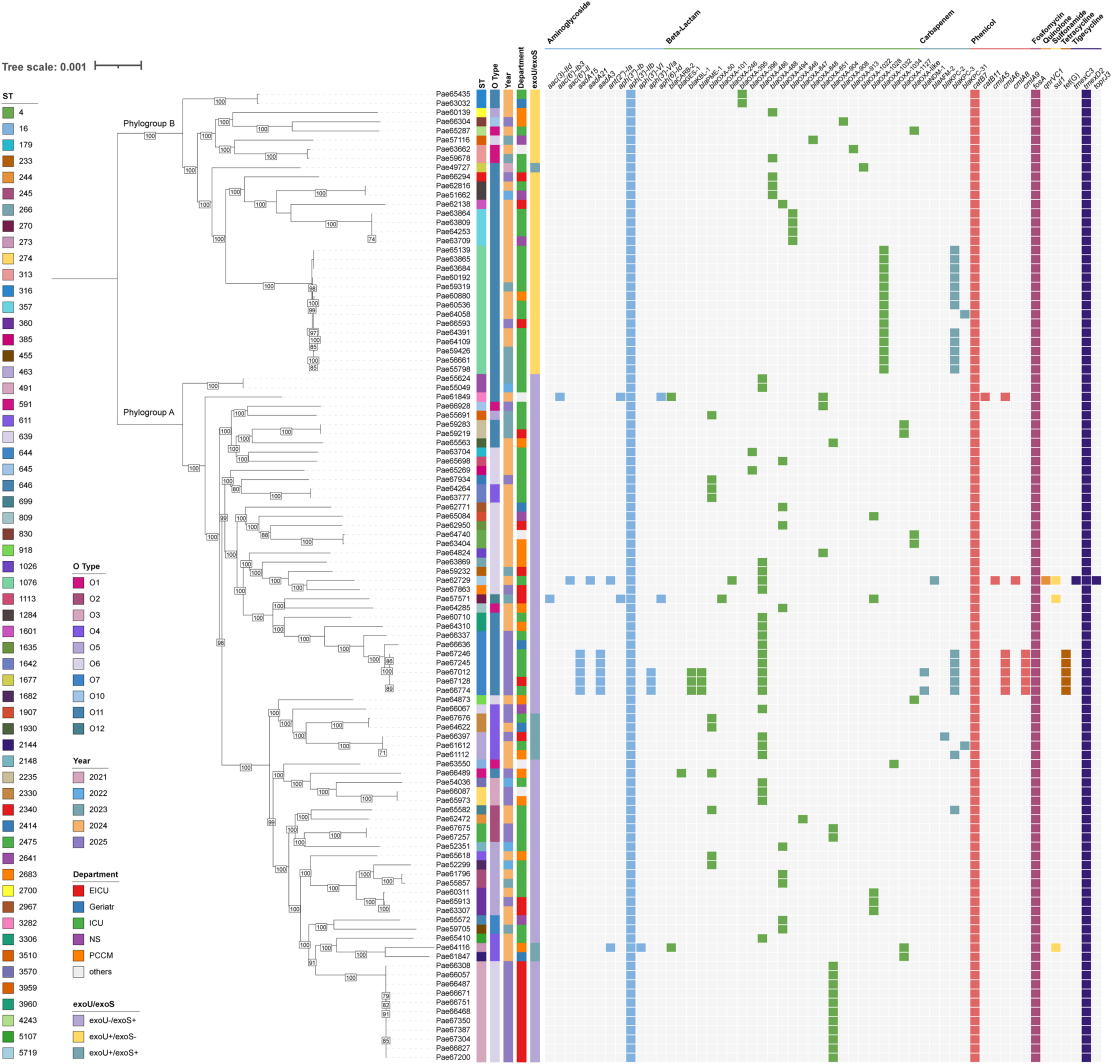
Phylogenetic tree and heatmap of annotations and clinical characteristics among 106 CRPA strains. A core-gene phylogeny(left) and cluster characteristics (right). FastTree of 106 CRPA strains was used to construct a phylogenetic tree based on the Maximum Likelihood method and further visualised using iTOLv5. The phylogeny featured two clusters were labelled as phylogroup-represented by the moderately virulent strain PAO1 and phylogroup B-exemplified by the highly virulent strain PA14. In the heatmap, The information of STs, O types, years, departments, and virulence genotypes was color-coded, with the corresponding legends provided on the far left. The fifth column to the left of the bacterial name corresponds to the *exoU*+/*exoS*-,*exoU*+/*exoS*- and *exoU*+/*exoS*- profile. The rightmost column indicates the presence (colored boxes) or absence (light gray boxes) of drug resistance genes. One-way analysis of variance and binary logistic regression were used to analyze the association between drug resistance gene and *exoU*+/*exoS*-,*exoU*+/*exoS*- and *exoU*+/*exoS*-.

This study identified a diverse array of resistance genes in the 106 CRPA strains. However, the detection rates for most genes were generally low. *Aph(3’)-IIb*, *catB7*, *fosA*, and *tmexD2* were the four most prevalent resistance genes, which were identified in all strains. Additionally, we identified 1 subtype of the *bla*_OXA-10_ family (*bla*_OXA-101_), 19 known subtypes of the *bla*_OXA-50_ family, and 2 novel subtypes of the *bla*_OXA-50_ family. It is worth noticing that a total of 23 strains (21.7%) were found to possess carbapenemase genes. Among these, 21 strains (19.8%) contained a single-carbapenemase gene, with *bla*_KPC-3_ being the perdominant type (17 strains). Other detected single-carbapenemase genes included *bla*_NDM-1_, *bla*_AFM-2_, *bla*_KPC-2_, and *bla*_KPC-31._ Furthermore, 2 strains co-carried *bla*_KPC-3_ and *bla*_NDM-1_. Furthermore, the mobile multidrug efflux pump gene cluster tmexC3-tmexD2-toprJ3 was identified in strain Pae62729 (**Figure 1**).

Further, univariate analysis was performed to compare *exoU*+/*exoS*- and *exoU*-/*exoS*^+^ group based on their clinical characteristics (age, length of hospital stay, exposure to≥3 antibiotics, ICU admission) and the presence of the resistant genes, the results showed that *exoU+/exoS-* strain exhibited a higher propensity to co-carry *bla*_KPC-3_ (12 strains vs. 6 strains), *bla*_OXA-488_ (5 strains vs. 0 strains), *bla*_OXA-846_ (4 vs 0), and *bla*_OXA-1032_ (14 vs 0) and a higher proportion of patients with ICU admission(22 vs 24). In contrast, *exoU*-/*exoS*+ strains were predominantly associated with *bla*_OXA-486_ (20 strains vs. 0 strains), and *bla*_OXA-904_ (14 strains vs. 0 strains)(**Table 3**). Subsequently, Benjamini-Hochberg procedure to control the false discovery rate (FDR) was applied, with significance defined as q < 0.05. *ExoU*+/*exoS*- group was likely to be significantly asscociated with the presence of ICU admission, *bla*_KPC-3,_*bla*_OXA-488,_*bla*_OXA-1032,_ while *exoU*-/*exoS*+ exhibited a significant association with the presense of *bla*_OXA-486,_*bla*_OXA-904,_ which was supported by FDR-corrected results (all q-values < 0.05) (**Table 3**).

**Table 3:** FDR-corrected results for resistance rene differences and clinical feature factors bwtween *exoU*+/*exoS*- and *exoU*-/*exoS*+ virulotypes.

Relevant factors	Value	95% comfidence interval		*P*	FDR
		Lower	Upper		
ICU admission	0.255	0.099	0.653	0.004	**0.0415**
*Bla*_KPC-3_	0.145	0.048	0.441	0.0005	**0.0094**
*Bla*_OXA-486_	a	a	a	0.0003	**0.0078**
*Bla*_OXA-488_	a	a	a	0.0021	**0.0283**
*Bla*_OXA-846_	a	a	a	0.0076	0.0522
*Bla*_OXA-1032_	a	a	a	4.5e-9	**2.475e-7**
*Bla*_OXA-904_	a	a	a	0.0046	**0.0423**

a:The *bla*_OXA-488_, *bla*_OXA-846_ and *bla*_OXA-1032_ genes were all distributed in group *exoU*+/*exoS*-, whereas the *bla*_OXA-486_ and *bla*_OXA-904_ were exclusively observed *exoU*+/*exoS*-, leading to highly imbalcanced data distribution. Thehefore, fisher’s exact test was employed to assess statistical significance, and the exact *P* value was reported. The value tended to infinity due to the zero cell problem and was not reported.

To control the false positive risk brought by multiple comparisons, the false discovery rate (FDR) correction was applied to all the P values obtained from the tests. Bold values indicated statistically significant differences after FDR correction (p < 0.05).

### 3.4 Mutation mechanisms of porin and efflux pump

**Figure 2:**
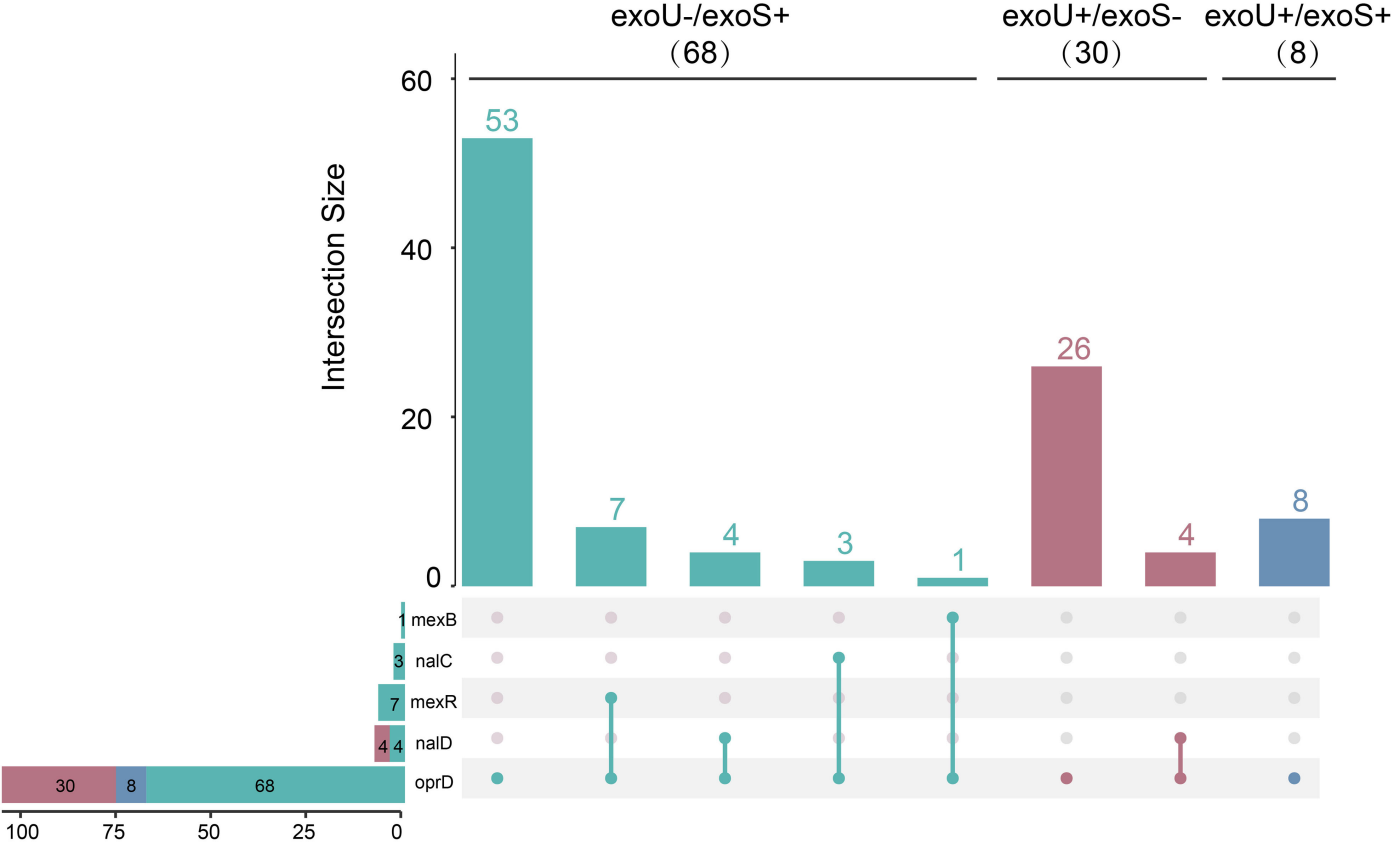
The intersection of membrane porin and efflux pump repressor mutant genes across strains with different virulence genotypes. The upset plot displays the number of shared membrane pore-forming and efflux pump repressor mutant genes across strain groups with distinct virulence genotypes, specifically *exoU*-/exoS+, *exoU*+/*exoS*-, *exoU*+/*exoS* +. The bar chart above indicates the total number of mutant genes in each virulence genotype group(set). The dot matrix connection chart below shows the pairwise intersections among the groups. A total of 106 clinical isolates’ data were included in this analysis.

Among 106 CRPA strains, the proportions of strains exhibiting mutations in the chromosomal genes *oprD*, *mexR*, *mexB*, *nalC*, and *nalD* were 100% (106/106), 6.6% (7/106), 0.9% (1/106), 3.8% (4/106) and 6.3% (8/106), respectively. Overall, 17.0% (18/106) of CRPA strains contained mutations in at least one of the three repressor genes associated with mexAB (*mexR*, *nalC*, and *nalD*). The predominant mutation type in the *oprD* gene was missense mutation (106/106), followed by insertions/deletions leading to frameshifts (33/106) and acquisition of stop codons (20/106). Amino acid alterations associated with the mutations are presented in **Table 4**. No statistically significant difference was observed across the mutation genes from *exoU*-/*exoS*+ to *exoU*+/*exoS*+ (**Figure 2**).

**Table 4:** Amino acid variation loci among matated genes of *oprD*, *mexR*, *mexB*, *nalC*, and *nalD* in carbapenem resistance *Pseudomonas aeruginosa* isolates from lower respiratory tract infections.

Mutated gene	Types	Amino acid variation loci
oprD	missense_variant	Asp43Asn, SerGlySer57GluGlyArg, Phe69Val, Thr103Ser, Gly106Asp, Lys115Thr, Val127Leu, Phe170Leu, GluPro185GlnGly, Val189Thr, Glu202Gln, Ile210Ala, Glu230Lys, Ser240Thr, Asn262Thr, Ala267Ser, Lys296Gln, Gln301Glu, Ala315Gly, Thr276Ala, Ser278Pro, Ala281Gly, Gln424Glu, Gly425Ala
	frameshift_variant	Ala15fs, Ser57fs, Ser59fs, Thr72fs, Asn107fs, Met111fs, Val127fs, Glu140fs, Phe148fs, Gly151fs, Gln158fs, Thr161fs, Gly162fs, Thr181fs, Thr188fs, Thr203fs, Ser223fs, Leu224fs, Arg234fs, Leu292fs, Val352fs, Gly314fs, Arg342fs, Gln402fs, Pro405fs, Gly314fs,
	stop_gained	Trp65*, Trp138*, Tyr196*, Trp277*, Trp339*, Gln415*
	splice_region_variant& stop_retained_variant	Ter444Ter
	start_lost	Met1?
	missense_variant& disruptive_inframe_deletion	Phe69del
mexR	frameshift_variant	Glu27fs, Lys44fs, Leu102fs, Leu123fs
	stop_gained	Gln25*, Gln31*
mexB	frameshift_variant	Asn700fs
nalC	disruptive_inframe_ deletion	Val115_, Ala116del
	conservative_inframe_deletion	Arg13_, Arg16del, Tyr129_, Gly132del
nalD	frameshift_variant	Thr11fs, Ile15fs, Gly41fs, Val42fs, Val151fs

### 3.5 Molecular epidemiology of strains

The MLST prediction results indicated that this population contained 59 distinct STs, demonstrating a high level of genetic diversity. Notably, we also identified the top ten global high-risk clones: 1 strain of ST233, 1 strain of ST244, and 4 strains of ST357. Additionally, ST1076 (14/106, 13.2%) and ST491 (11/106, 10.4%) were the most prevalent types; however, they were not dominant within the overall population, which exhibited a highly dispersed distribution of STs. 10 O-antigen serotypes were identified through O-antigen serotypes, with O11 (41/106, 38.7%) and O6 (27/106, 25.5%) as the most prevalent. ST1076-O11 (13/106, 12.3%) was predominantly found in ICU, whereas ST493-O6 (11/106, 10.4%) was solely identified in EICU. Three strains of the potentially high-risk ST463-O4 were sporadically distributed across the ICU, EICU, and Geriatrics Department.

Based on the distribution of virulence genes *exoU* and *exoS*, the phylogenetic tree exhibited distinct population differentiation in its structure: the upper clade was predominantly assigned to phylogroup B (characterized by *exoU*+/*exoS*- and exemplified by the highly virulent strain PA14); the lower clade was predominantly linked to phylogroup A, characterized by *exoU*-/*exoS*+ and represented by the moderately virulent strain PAO1. Among 106 CRPA strains, the quantity of phylogroup A strains was significantly greater than that of phylogroup B strains. *exoU*+/*exoS*- strain was primarily identified as ST1076 (14/30, 46.7%) and ST357 (4/30, 13.3%). Conversely, *exoU*-/*exoS*+ strains exhibited a broader ST distribution, encompassing up to 43 types, with ST491 (11/68, 16.2%) and ST644 (7/68, 10.3%) being the most common. The *exoU*+/*exoS*+ strains were dominated by ST463-O4 (3/8, 37.5%) (**Figure 1**).

### 3.6 Survival rate of *Galleria mellonella* post-infection

**Figure 3:**
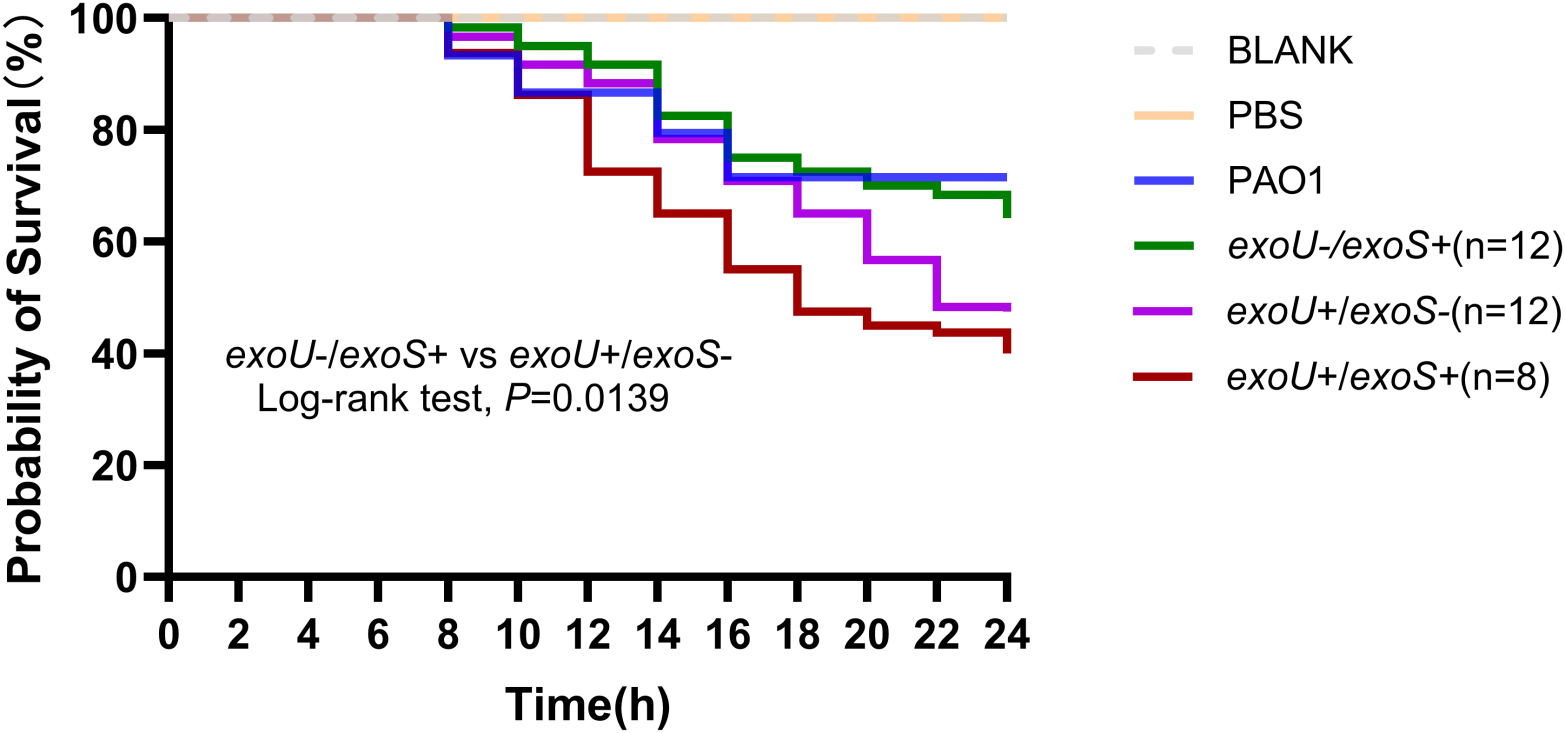
Survival curves of *Galleria mellonella* larvae following CRPA infection. The *in vivo* virulence of different virulence genotypes strains was verified using a galleria mellonella larva infection model, with *Pseudomonas aeruginosa* PAO1 as the median virulent control and no treatment and sterile phosphate buffered saline (PBS) as the blank control. Bacteria (1×10^4^ CFU/mL) were injected into the larvae, and the survival curves were monitored for 24 hours. The numbers of *exoU*-/*exoS*+, *exoU*+/*exoS*-, and *exoU*+/*exoS*+ strains were 12, 12, and 8, respectively. The K–M survival curves among *exoS*+/*exoU*-, *exoS*-/*exoU*+, and *exoS*+/*exoU*+ (*exoS*+/*exoU*- vs *exoS*-/*exoU*, *P* = 0.0139). The *P* value of Kaplan Meier’analysis was calculated with log-rank test.

We conducted an analysis of the virulence associated with all 8 strains from *exoU*+/*exoS*+ and 12 strains each from *exoU*-/*exoS*+ and *exoU*+/*exoS*-. The analysis revealed that the mean 24h death rates for the control groups (BLANK and PBS group), PAO1, *exoU*-/*exoS*+, *exoU*+/*exoS*-, *exoU*+/*exoS*+ group were 0%, 0%, 40.0%, 35.9%, 52.5%, 60.0%, respectively. Statiscally significant difference was observed between *exoU*-/*exoS*+ and *exoU*+/*exoS*-(*P* = 0.0139) (**Figure 3**). These findings indicated that the clinical isolates of such as Pae55798 (ST1076-O11, Pae61612(ST463-O4), Pae63709(ST357-O11), exhibited higher virulence, significant drug resistance. Further, comparisons between individual strains indicated an overlap in death rate among these different virulence gene phenotypes (**Supplementary Table 3**).

## 4 Discussion

According to the Global Burden of Disease (GBD) 2019, lower respiratory tract infections were the leading cause of infection-related mortality. More than 4 million people died worldwide from these infections in that year, and they became the infection syndrome with the highest mortality in the five super-regions (GBD 2019 Antimicrobial Resistance Collaborators, 2022). *Pseudomonas aeruginosa* is one of the important pathogenic bacteria in this field. It has strong drug resistance and poses treatment challenges, frequently resulting in serious complications, including ventilator-associated pneumonia. It poses a life threat to patients with pre-existing conditions and people with compromised immune systems. In addition, *Pseudomonas aeruginosa* exhibits a greater propensity for mutation and possesses more intricate drug resistance mechanisms compared to other gram-negative bacteria (Yang et al., 2025). Currently, there is a concerning global increase in *Pseudomonas aeruginosa* that produce KPC enzymes and isolates that carry the *exoU* and *exoS* virulence genes (Zhang et al., 2022; Zhang et al., 2022; Sun et al., 2023; Li et al., 2024). These strains have raised significant concern and vigilance due to their extensive drug resistance and high virulence.

In our study, all strains were found to carry the genes *aph(3’)-IIb*, *catB7* and *fosA*, which are related to resistance to aminoglycosides, chloramphenicol and fosfomycin. The presence of these three genes indicated the potential existence of integrons. In addition, we detected the presence of various β-lactamases, including carbapenemases. Epidemiological data indicated that the majority of infected patients had prior exposure to β-lactamase inhibitors, specifically piperacillin/tazobactam before the detection of *bla*_OXA-50_ strains. The MIC for the strains against piperacillin/tazobactam exhibited a general increase, with a drug resistance rate of 45.2%, which is close to the values reported in Japan and other countries (Yano et al., 2024). Traditional β-lactamase inhibitors exhibited limited inhibitory activity against *bla*_OXA-50_*(*Horcajada et al., 2019). We detected 19 known subtypes of the *bla*_OXA-50_ family and identified 2 new unknown subtypes. These findings together suggest that the *bla*_OXA-50_ family may undergo functional differentiation through continuous genetic evolution and play a key role in the development of piperacillin/tazobactam resistance phenotypes. The extensive application of β-lactamase inhibitors may serve as a selective pressure, influencing the adaptive evolution and subtype diversification within this gene family. This mechanistic hypothesis offers a significant theoretical foundation for the optimization of inhibitors and the formulation of drug resistance monitoring strategies. Various carbapenemases have been identified in CRPA isolates across different geographical regions. Isolates from South America and Central America predominantly carried *bla*_KPC-2_ and *bla*_VIM-2_; *bla*_NDM-1_ and *bla*_IMP-1_ represent the predominant carbapenemase genes identified in Australia and Singapore (Reyes et al., 2023). However, *bla*_KPC-3_ was found as the predominant enzyme type in this study, with only a single strain of *bla*_KPC-2_ identified. This finding contrasts sharply with previous reports from China, where *bla*_KPC-2_ was typically the main enzyme type observed (Reyes et al., 2023). Although some strains were resistant to carbapenems, these strains remained susceptible *in vitro* to other β-lactams, cephalosporins, or fluoroquinolones. In such cases, traditional β-lactams should be prioritized to avoid the utilization of new antibiotics. We also noticed a significant proportion of DTR-PA. Numerous studies have confirmed that ceftazidime-avibactam (CZA) exhibited substantial effectiveness against clinical strains of *Pseudomonas aeruginosa* responsible for lower respiratory tract infections, including DTR-PA (Corbella et al., 2022; Shields et al., 2025). However, the number of CZA-resistant strains is also increasing. In this study, the resistance rate to CZA reached 10%, which was lower than the 18.1% reported by the China Antimicrobial Surveillance Network in 2024 (CHINET.CRPA., 2025). CZA resistance is linked to: (I) mutations in β-lactamases, such as KPC, AmpC, CTX-M, and OXA-48; (II) the production of metallo-β-lactamases that are not inhibited by avibactam; (III) overexpression of hydrolases; (IV) heightened efflux pump activity, including AcrA/B–TolC and MexA/B–OprM; and (V) diminished cellular permeability (Horcajada et al., 2019). The high prevalence of KPC-3 in our study further explained the CZA resistance observed in certain strains, as previous research has documented the D179Y substitution in the Ω-loop of KPC-3, resulting in the KPC-31 variant linked to CZA resistance (Faccone et al., 2022). Therefore, laboratories should actively conduct *in vitro* AST of CZA, and clinicians should maintain vigilance regarding KPC mutations during CZA administration.

Previous studies have reported that the loss or mutation of *oprD* was associated with imipenem resistance. In contrast, the mechanisms of meropenem resistance exhibit greater diversity (Fratoni et al., 2024; Yano et al., 2024). Herein, we demonstrated that all strains examined harbored mutations within the *oprD* gene, and the majority of the amino acid alteration at these loci resulting from nonsense mutions have been previously documented to induce varying degrees of functional alterations in oprD (Nazari et al., 2023; Wang et al., 2025). Mutation in the *oprD* of *Pseudomonas aeruginosa* could enhance the adaptability of the bacterium to the host environment during infection (Skurnik et al., 2013). Additionally, other study has reported that inactivating mutations in oprD could arise even in the absence of carbapenems exposure (Wolter et al., 2008), suggesting that the loss of oprD protein function may confer a selective survival advantage. Additionally, mutations associated with efflux pump regulation constituted a comparatively minor proportion, even less than those related to acquired carbapenemase-mediated resistance mechanisms. However, it is challenging to establish a direct causal link because every strain possesses a variety of resistance mechanisms. Future studies will aim to further validate this hypothesis through *in vitro* and *in vivo* functional assays.

T3SS constitutes the fundamental component of the pathogenic mechanism in *Pseudomonas aeruginosa*. It functions as a “molecular syringe,” facilitating the direct delivery of toxins into host cells, and secretes four characteristic effector proteins: *exoS*, *exoT*, *exoY*, and *exoU*. In this study, we focused on *exoS* and *exoU*, which are key factors in the pathogenicity of *Pseudomonas aeruginosa*, and explored the association among different genotypic patterns, resistance to common antibacterial agents, and the mechanisms underlying this resistance. Our study found that the detection rate of *exoS*+/*exoU*- was the highest, aligning with the findings of Sarges, and Tang et al (Sarges et al., 2020; Tang et al., 2025). In addition, our findings indicated that *exoU*+/*exoS*- strains exhibited an increased resistance rate to ceftazidime, cefepime, and piperacillin/tazobactam. Previous study (Palzkill, 2018) has reported that KPC-3 showed increased kcat/KM values for ceftazidime hydrolysis compared to KPC2, therefore we speculate that a higher rate of drug resistance may be associated with the presence of these strains alongside certain subtypes of *bla*_KPC-3_. We found that *exoU*+/*exoS*- group was likely to be significantly asscociated with the presence of ICU admission, *bla*_KPC-3_, *bla*_OXA-488_, *bla*_OXA-1032_, while *exoU*-/*exoS*^+^ exhibited a significant association with the presense of *bla*OXA-486, *bla*OXA-904. These associated phenomena are trends worthy of attention, which provide clues for further research of the distribution of bacterial characteristics in specific cinical settings. Further, our analysis revealed that *exoU*, *exoS* and the aforementioned drug resistance genes were located on distinct scaffolds, and no structural association between them was supported by the current evidence. Nevertheless, this does not exclude the possibility that they may reside together on a single genetic element(such as a plasmid) that has not been fully assembled due to technical limitations of next-generation sequencing scan map. The fragmented nature of second-generation sequencing assemblies may result in the physical separation of co-located sequences across different scaffolds, thereby precluding reliable inference of their linkage. These speculations need to be confirmed through further experiments such as third-generation sequencing, thereby synergistically enhancing clinical adaptability.

The analysis of MLST and O-antigen serotypes revealed notable characteristics related to the population genetic structure and the distribution of virulence genes. The analysis of MLST and O-antigen serotypes revealed notable characteristics related to the population genetic structure and the distribution of virulence genes. Notably, the identification of high-risk clones ST233, ST244, and ST357 (Del Barrio-Tofiño et al., 2020) indicated the potential presence of evolutionary selection pressures associated with antimicrobial resistance or host adaptability within the hospital environment. While ST1076 and ST491 were relatively predominant, they did not establish a single dominant clone within the population, suggesting that the transmission of current strains may demonstrate a polyclonal nature. O-antigen serotypes were dominated by O11 and O6. The concentrated distribution of ST1076-O11 in ICU and the exclusive detection of ST493-O6 in EICU established a specific spatial distribution pattern. This may be closely related to variations in device usage patterns, antibiotic exposure, or patient immune status across different ICU. In this study, the majority of O11 strains were found to carry *exoU* and were predominantly DTR-PA. The direct involvement of the O11 antigen in the pathogenic process has yet to be conclusively established through gene knockout experiments, and the existing data do not exclude the possibility of it serving solely as a concomitant marker. It is worth noticing that the ST463 CRPA clone carrying the *bla*_KPC_, *exoU*, and *exoS* genes has emerged and spread in East China (Hu et al., 2021; Li et al., 2024). The dispersed distribution of these strains within our hospital indicates a necessity for vigilance to mitigate the risk of cross-ward transmission through healthcare professionals or equipment.

The analysis of virulence gens showed a distinct differentiation between phylogroup A (*exoS*+) and phylogroup B (*exoU*+), which is consistent with the evolutionary patterns observed in PAO1 (phylogroup A) and PA14 (phylogroup B). Although phylogroup A strains were significantly predominant in number, the clustering of ST1076 and ST357 in phylogroup B may reflect that these ST strains have enhanced their invasive virulence through the acquisition of *exoU* in T3SS. The *Galleria mellonella* larva infection study further validated the high-virulence potential of ST1076, ST463, and ST357 strains carring *exoU*. This aligns with previous studies reporting a correlation between *exoU*+ strains and unfavorable outcomes in severe pneumonia (Hauser et al., 2002). However, in this model, we also observed different virulence levels within these three virulence genotypes, indicating that the factors influencing the virulence of CRPA are multifaceted. These factors may not only include the type and quantity of virulence genes carried by the strains but also their expression levels (Oliveira et al., 2021), *G. mellonella* has been used to study the virulence treatment of a wide range of microorganisms, including *P. aeruginosa* (Asai et al., 2023; Campo-Pérez et al., 2025), but this model still has certain limitations. Firstly, the lack of a standardized supplier for genetically uniform larvae introduces variability in experimental outcomes. We observed significant differences in mortality rates across different batches when exposed to the same bacterial strain, suggesting batch-to-batch inconsistency that may affect reproducibility. In addition, it may not accurately reflect the pathogenicity of the pathogen in mammalian hosts. This may be related to the essential differences in the immune system between *G. mellonella* and mammals, which leads to the inability of some virulence factors of the pathogen to be fully expressed or function in the model (Russo and MacDonald, 2020). Therefore, the results of this study mainly indicate the relative virulence of these strains, and their direct association with human clinical outcomes still needs to be further confirmed. The diversity of ST types (43 types) among *exoU*-/*exoS*+ strains indicated that the anti-phagocytic effect mediated by *exoS* (Sawa et al., 2014) was broadly preserved across strains with varying genetic backgrounds. The enrichment of ST463 in the eight *exoU*+/*exoS*+ double-positive strains was noteworthy, which might indicate that this ST type had acquired an additional virulence module via horizontal gene transfer (Song et al., 2023). This rare phenotype holds considerable importance in clinical practice, with studies indicating that the 28-day mortality rate for patients infected with ST463 *exoU*+/*exoS*+ strains was significantly elevated compared to the non-ST463 group (Hu et al., 2021). These data suggest that ST463 may evolve into a significant clinical threat.

In summary, this study offers novel insights for enhancing clinical infection control of CRPA and advancing laboratory detection methods. The prevalence trend of specific clones (e.g., ST1076-O11) in high-risk wards such as the ICU suggested that environmental microbial monitoring needs to be strengthened; the potential association between phylogroup B strains (*exoU*+/*exoS*- type) represented by the highly virulent PA14 and invasive infections necessitated the implementation of rapid classification protocols based on virulence genotypes in clinical laboratories. However, this study also presents several limitations: Firstly, our samples exclusively encompassed CRPA derived from lower respiratory tract sources. Secondly, although we observed that the co-occurrence of the *exoU* gene with resistance genes (e.g., *bla*_KPC-3_) and some subtypes of the *bla*_OXA-50_ gene, the mechanisms underlying these observations remain inadequately investigated. In the future, further studies should incorporate multicenter samples of various infection types. A thorough analysis of the three-dimensional association mechanisms among genetic lineages, virulence characteristics, and clinical outcomes should be analyzed in depth combined with clinical course data.

## Data Availability

The datasets presented in this study can be found in online repositories. The names of the repository/repositories and accession number(s) can be found in the article/**Supplementary Material**.
