# Supplementary material for: Molecular epidemiological association between antimicrobial resistance characteristics and type III secretion system in carbapenem-resistant Pseudomonas aeruginosa from lower respiratory tract infections
Source: Front Cell Infect Microbiol. 2026 Feb 17;16:1757868. doi: 10.3389/fcimb.2026.1757868 (PMC12953427; doi:10.3389/fcimb.2026.1757868)
Supplement: Supplementary file 2 [file Table2.docx]

Supplementary Table 2. Resistance rate (percentages), minimum inhibitory concentrations (MICs) of Carbapenem resistance *Pseudomonas aeruginosa* isolates from lower respiratory tract infections

| Antimicrobial agents | *Pseudomonas aeruginosa*(n=106) | | | | |
| --- | --- | --- | --- | --- | --- |
|  | S | I | R | MIC50  (ug/mL) | MIC90  (ug/mL) |
| Ceftazidime | 59 | 7 | 40 | 8 | >128 |
| Cefepime | 61 | 18 | 27 | 8 | >128 |
| Aztreonam | 30 | 37 | 39 | 16 | >64 |
| Tobramycin | 94 | 2 | 10 | 0.5 | 2 |
| Ciprofloxacin | 31 | 74 | 1 | 1 | 16 |
| Levofloxacin | 32 | 27 | 47 | 2 | 16 |
| Imipenem | 1 | 7 | 98 | 8 | >128 |
| Meropenem | 13 | 19 | 74 | 8 | >128 |
| Piperacillin/tazobactam | 53 | 5 | 48 | 32 | >128 |
| Cefoperazone/sulbactam | 54 | 19 | 33 | 0.5 | >128 |
| Ceftazidime/avibactam | 96 | 0 | 10 | 2 | 8 |
| Polymyxin B | 106 | 0 | 0 | 1 | 1 |

S, susceptible; I, intermediate susceptibility; R, resistant
